# Annotated Draft Genome Assemblies for the Northern Bobwhite (*Colinus virginianus*) and the Scaled Quail (*Callipepla squamata*) Reveal Disparate Estimates of Modern Genome Diversity and Historic Effective Population Size

**DOI:** 10.1534/g3.117.043083

**Published:** 2017-07-17

**Authors:** David L. Oldeschulte, Yvette A. Halley, Miranda L. Wilson, Eric K. Bhattarai, Wesley Brashear, Joshua Hill, Richard P. Metz, Charles D. Johnson, Dale Rollins, Markus J. Peterson, Derek M. Bickhart, Jared E. Decker, John F. Sewell, Christopher M. Seabury

**Affiliations:** * Department of Veterinary Pathobiology, College of Veterinary Medicine and Biomedical Sciences, Texas A&M University, College Station, Texas 77843; † Department of Veterinary Integrative Biosciences, College of Veterinary Medicine and Biomedical Sciences, Texas A&M University, College Station, Texas 77843; ‡ Genomics and Bioinformatics Core, Texas A&M AgriLife Research, College Station, Texas 77843; § Rolling Plains Quail Research Foundation, Rotan, Texas 79546; ** Department of Biological Sciences, Border Biomedical Research Center, University of Texas at El Paso, Texas 79968; †† Dairy Forage Research Center, United States Department of Agriculture–Agricultural Research Service, Madison, Wisconsin 53706; ‡‡ Division of Animal Sciences, University of Missouri, Columbia, Missouri 65211; §§ Harris Ranch, Uvalde, Texas 78801

## Abstract

Northern bobwhite (*Colinus virginianus*; hereafter bobwhite) and scaled quail (*Callipepla squamata*) populations have suffered precipitous declines across most of their US ranges. Illumina-based first- (v1.0) and second- (v2.0) generation draft genome assemblies for the scaled quail and the bobwhite produced N50 scaffold sizes of 1.035 and 2.042 Mb, thereby producing a 45-fold improvement in contiguity over the existing bobwhite assembly, and ≥90% of the assembled genomes were captured within 1313 and 8990 scaffolds, respectively. The scaled quail assembly (v1.0 = 1.045 Gb) was ∼20% smaller than the bobwhite (v2.0 = 1.254 Gb), which was supported by kmer-based estimates of genome size. Nevertheless, estimates of GC content (41.72%; 42.66%), genome-wide repetitive content (10.40%; 10.43%), and MAKER-predicted protein coding genes (17,131; 17,165) were similar for the scaled quail (v1.0) and bobwhite (v2.0) assemblies, respectively. BUSCO analyses utilizing 3023 single-copy orthologs revealed a high level of assembly completeness for the scaled quail (v1.0; 84.8%) and the bobwhite (v2.0; 82.5%), as verified by comparison with well-established avian genomes. We also detected 273 putative segmental duplications in the scaled quail genome (v1.0), and 711 in the bobwhite genome (v2.0), including some that were shared among both species. Autosomal variant prediction revealed ∼2.48 and 4.17 heterozygous variants per kilobase within the scaled quail (v1.0) and bobwhite (v2.0) genomes, respectively, and estimates of historic effective population size were uniformly higher for the bobwhite across all time points in a coalescent model. However, large-scale declines were predicted for both species beginning ∼15–20 KYA.

Annual declines of ≥3% in the estimated abundance of northern bobwhite (*Colinus virginianus*; hereafter bobwhite) and scaled quail (*Callipepla squamata*) have been noted since at least 1996 (Sauer *et al.* 2014). The New World quail is a family (Odontophoridae) comprised of over 30 species of ground-dwelling birds ranging across much of the Americas, from open deserts to tropical forests (Madge *et al.* 2002; Carroll *et al.* 2016). The historic range of the widely distributed and phenotypically diverse bobwhite overlaps to some degree with that of the more phenotypically uniform scaled quail in the semiarid southwestern United States (Madge *et al.* 2002; Carroll *et al.* 2016). The natural abundance of the scaled quail and the bobwhite are both known to follow boom-bust population patterns, rapidly expanding during years with favorable conditions, and declining sharply given poor conditions, thereby further complicating long-term conservation efforts when considered within the context of recurrent annual declines (Peterson 2001; Hernandez and Peterson 2007; Silvy *et al.* 2007).

Hypotheses regarding the potential origins of bobwhite and scaled quail declines have often pointed to an array of complex interactions between various environmental and human factors including weather, habitat fragmentation or loss, predation, disease, toxins, and harvest intensity, particularly during a drought (Clawson and Baker 1959; Wilson and Crawford 1988; Brennan 1991, 1994; Bridges *et al.* 2001; Rollins and Carroll 2001; Williams *et al.* 2004; Hernández *et al.* 2013; Dunham *et al.* 2014). However, most quail biologists agree that habitat loss, fragmentation, and degradation are primary causes of bobwhite and scaled quail declines (Roseberry and Klimstra 1984; Peterson 2001; Williams *et al.* 2004; Tomeček *et al.* 2015). Despite predation by various animals including humans (*i.e.*, hunting) being a leading cause of quail mortality, and likely influencing bobwhite numbers more markedly than those of scaled quail, predation is unlikely to have caused broad-scale and long-term declines (Brennan 1994; Rollins and Carroll 2001; Hernandez and Peterson 2007; Silvy *et al.* 2007; Tomeček *et al.* 2015; Peterson 2001). Moreover, infectious and parasitic diseases (*i.e.*, eyeworms, *Oxyspirura petrowi*; avian poxvirus; West Nile Virus), as well as environmental toxins (*i.e.*, heptachlor and dieldrin; now banned in the United States of America) have also been suggested as potential factors influencing more recent quail population declines (Clawson and Baker 1959; Wilson and Crawford 1988; Reed and Schrader 1989; Syracuse Research Corporation 2002, 2007; Komar *et al.* 2003; Dunham *et al.* 2014). However, no specific infectious or parasitic diseases, including eyeworms, have been shown to be causal for broad-scale declines among bobwhites or scaled quail (Peterson 2007).

Several recent studies have sought to develop tools and resources for large-scale population studies in the bobwhite, including the development of a cDNA microarray, a first-generation draft genome assembly, and several mitochondrial population genomics studies (Rawat *et al.* 2010; Halley *et al.* 2014, 2015; Williford *et al.* 2014a,b). However, no draft genome assembly currently exists for the scaled quail. The continuous evolution of sequencing technologies (*i.e.*, higher yields and cheaper costs) and publicly available genome assembly algorithms provides a natural segue to the development of a first-generation scaled quail draft genome assembly, and a second-generation assembly for the bobwhite.

Herein, we present draft genome assemblies with annotation for the scaled quail (first-generation) and the bobwhite (second-generation), and use these resources to comparatively estimate historic effective population sizes for both species, as inferred from modern estimates of genome diversity. The second-generation bobwhite draft genome assembly markedly improves contiguity, with a 45-fold improvement in N50 scaffold size, as compared to the first-generation assembly, which aggressively sought to suppress structural assembly errors (Halley *et al.* 2014). We also assess genome completeness and annotation using BUSCO (Simão *et al.* 2015), with comparison of both quail draft genome assemblies to other well-established avian genomes. Finally, we also employed the whole genome shotgun sequence detection method (WSSD) to comparatively investigate the frequency and distribution of segmental duplications within the scaled quail and bobwhite genomes. The resources presented in this study are expected to further catalyze interdisciplinary research programs focusing on modern bobwhite and scaled quail populations.

## Materials and Methods

### DNA isolation and genome sequencing strategy

Genomic DNA isolated from the legs (skeletal muscle) of a wild, lawfully harvested female bobwhite (Central Great Plains ecoregion of Texas) was available from previous studies (Halley *et al.* 2014, 2015). Likewise, we isolated genomic DNA from skeletal muscle acquired from the legs of a wild, adult female scaled quail (named “Morgan”), which was lawfully harvested in the Southern Texas Plains ecoregion. Ethical clearance is not applicable to samples obtained via lawful harvest. Briefly, scaled quail skeletal muscle from the thigh and lower legs was resected and minced using a scalpel, and genomic DNA was isolated using the Epicentre MasterPure DNA purification kit (Cat. No. MC85200), as recommended by the manufacturer (Epicentre Biotechnologies Inc., Madison, WI). DNA yield was initially quantified using a NanoDrop 1000 (NanoDrop Technologies Inc., Wilmington, DE), and the presence of high molecular weight genomic DNA was subsequently verified by agarose gel electrophoresis. For both the bobwhite and the scaled quail, small insert paired-end (PE) libraries were constructed using the TruSeq Nano LT Library Prep Kit (#FC-121-4001; Illumina, San Diego, CA), according to the standard protocol provided by Illumina. One PE library targeting 200 bp fragments was constructed for the bobwhite, and three PE libraries targeting 200, 300, and 600 bp fragments were constructed for scaled quail. All PE libraries were processed using PE-125 cycle runs (2 × 125 bp), with data generation carried out on an Illumina HiSeq 2500v4 High Output instrument. Additionally, two mate-pair (MP) libraries (4 and 8 kb) for the bobwhite, and four MP libraries (4, 5, 8, and 11 kb) for the scaled quail were constructed using the Nextera Mate Pair Library Prep Kit (#FC-132-1001; Illumina), and similarly processed (2 × 125 bp) on an Illumina HiSeq 2500v4 High Output instrument. The insert size distributions for all PE and MP libraries were estimated and visualized using the CLC Genomics Workbench 8.5.1 (https://www.qiagenbioinformatics.com/), as previously described (Halley *et al.* 2014). Given the absence of knowledge regarding specific properties of the scaled quail genome at the time of project initiation, we generated a more diverse set of PE and MP libraries for this species, as a means to ensure genome assembly using Illumina short read technologies.

### Genome assembly and assessment of completeness

The scaled quail genome (v1.0) and the second-generation bobwhite genome (v2.0) were both assembled using MaSuRCA version 2.3.2, with implementation of an updated scaffolder made available in version 3.2.0 for the bobwhite (Zimin *et al.* 2013). Within the MaSuRCA pipeline, we utilized the QuorUM error corrector for Illumina reads, and the Celera assembler after estimating the genome size for both quail species via Jellyfish 2.0 (Zimin *et al.* 2013; Marçais *et al.* 2015). K-mer length was set to “auto” for both genomes, and k-mer sizes of 86 and 87 were chosen for the scaled quail and bobwhite graphs, respectively. Jump coverage was limited to 300 for both assemblies, and the Celera Assembler settings within MaSuRCA were as recommended by the developer (*i.e.*, ovlMerSize = 30; cgwErrorRate = 0.14 ovlMemory = 4 GB). We also instructed MaSuRCA not to trim long runs of homopolymers. In summary, MaSuRCA assembled both genomes using reduced super-reads, paired-end linking mates, and filtered, deduplicated mate-pairs, with final gap closing as previously described (Miller *et al.* 2008, 2010; Zimin *et al.* 2013). Herein, we report on the scaffolds ≥1 kb for both the scaled quail and the bobwhite.

BUSCO (version 1.1b1) was used to quantitatively assess the completeness of the scaled quail (v1.0) and bobwhite (v2.0) draft genome assemblies (Simão *et al.* 2015). BUSCO, which relies on the programs tblastn, Augustus, and HMMER3 to investigate orthologous genes present within a genome assembly, was used in conjunction with 3023 vertebrate single copy orthologs to evaluate the scaled quail and bobwhite draft genome assemblies, and their corresponding annotation gene sets (Altschul *et al.* 1990; Stanke and Morgenstern 2005; Finn *et al.* 2011; Simão *et al.* 2015). For comparison, we performed identical runs of BUSCO on *Taeniopygia guttata* (Zebra Finch) 3.2.4 and *Gallus gallus* (Chicken) 4.0 downloaded from Ensembl (ensembl.org), *G. gallus* 5.0 downloaded from NCBI (ncbi.nlm.nih.gov/assembly), Turkey (*Meleagris gallopavo*) 5.0 downloaded from NCBI (ncbi.nlm.nih.gov/assembly), and 21 additional high coverage avian genomes downloaded from NCBI and the Nodai Genome Research Center (http://www.nodai-genome.org/japanese_quail.html?lang=en) (Huang *et al.* 2013; Kawahara-Miki *et al.* 2013; Shapiro *et al.* 2013; Zhang *et al.* 2014). The Augustus chicken model within BUSCO was used for all BUSCO analyses.

### Repetitive content and variant prediction

RepeatMasker (version 4.0.3; 20130422) was used in a two-step approach to estimate the minimum repetitive content within the scaled quail (v1.0) and bobwhite (v2.0) draft genome assemblies (Smit *et al.* 1996; Jurka *et al.* 2005). Briefly, both genomes were initially processed using the *G. gallus* repeat library, and thereafter, the resulting masked genomes were again processed using the *T. guttata* repeat library, as previously described (Seabury *et al.* 2013). Additionally, we used PHOBOS (version 3.3.12) to predict microsatellites within the scaled quail (v1.0) and bobwhite (v2.0) genomes, with specific parameters as follows: repeat unit size range was set from 2 to 10; Maximum successive N’s per repeat = 2; Recursion depth = 5; Minimum and maximum percentage perfection = 80 and 100% (Mayer *et al.* 2010). Published bobwhite and scaled quail microsatellites (Schable *et al.* 2004; Faircloth *et al.* 2009; Orange *et al.* 2014) were aligned to both genome assemblies using blastn.

Following a two-stage RepeatMasker analysis (*i.e.*, *G. gallus* + *T. guttata* repeat libraries), the masked scaled quail (v1.0) and bobwhite (v2.0) draft genome assemblies were used as the reference sequences for reference mapping and variant prediction [*i.e.*, single nucleotide variants (SNVs), multi-nucleotide variants (MNVs), insertion-deletion mutations (indels)], as previously described (Sánchez *et al.* 2009; Seabury *et al.* 2011, 2013). Genome-wide variant prediction analyses were performed using the CLC probabilistic variant detection algorithm (v8.5.1) as follows: Ignore broken read pairs = no; Ignore nonspecific matches = yes; Variant probability ≥0.95; Variant required in forward and reverse reads = yes; Minimum coverage ≥4 (Halley *et al.* 2014).

### Annotation

The MAKER annotation pipeline (version 2.31.8) was used to annotate the scaled quail (v1.0) and bobwhite (v2.0) draft genome assemblies (Cantarel *et al.* 2008). We utilized alternate species cDNA evidence from *G. gallus* 4.0, *M. gallopavo* 2.01, and *T. guttata* 3.2.4 derived from Ensembl (ensembl.org). Moreover, we used cDNA evidence from *C. virginianus* as alternate evidence for the scaled quail, and species-specific evidence for the bobwhite (Rawat *et al.* 2010; Halley *et al.* 2014). Ensembl (ensembl.org) protein homology evidence from *G. gallus* 4.0, *T. guttata* 3.2.4, and *M. gallopavo* 2.01 was also provided to the MAKER annotation pipeline. The RepeatMasker parameter in MAKER was set to all organisms, which facilitated the masking of repeats prior to annotation, and the Augustus gene prediction species model was set to chicken (*G. gallus*). EST2genome and Protein2genome settings were both set to 0. Postprocessing of the gff3 annotation files included MAKER protocols for assigning putative gene function using blastp, and the renaming of genes with the locus tag prefix assigned by NCBI WGS. These analytical processes were performed using the MAKER provided scripts maker_map_ids, map_gff_ids, and maker_functional_gff (Campbell *et al.* 2014). The UniProt/Swiss-Prot database used for the blastp putative gene function search consisted of 551,705 sequences, as downloaded on July 19, 2016. Parameter settings included evalue = 0.000001, num_alignments = 1, seg = yes, soft_masking = true, lcase_masking, and max_hsps = 1, according to the MAKER suggested protocol (Camacho *et al.* 2009; Campbell *et al.* 2014; The UniProt Consortium 2015).

### Segmental duplication and deletion analysis

WSSD analyses using JaRMS (https://github.com/njdbickhart/JaRMS) and RAPTR-SV (Bickhart *et al.* 2015) were performed on the scaled quail (v1.0) and bobwhite (v2.0) genomes to estimate the frequency and distribution of regions exhibiting putative evidence of segmental duplication and/or deletion, as inferred by read depth. JaRMS is a java-based port of the CNVnator algorithm (Abyzov *et al.* 2011) that automates analysis and is tolerant of smaller input contigs—as are typically generated by *de novo* assemblies. Identical to the CNVnator pipeline, JaRMS created a set of nonoverlapping, 500 bp windows for each scaffold, and thereafter, calculated global mean and SD from a fitted Gaussian distribution. Raw read depth in each window was then transformed using the same GC correction algorithm used by CNVnator (Abyzov *et al.* 2011). We set a duplication cutoff read depth at the average +3 SD and a deletion cutoff read depth at the average −2 SD as previously described (Alkan *et al.* 2009). Structural variants were called for specific genomic regions if six out of seven consecutive windows had read depths higher or lower than the cutoffs. We removed deletion windows that intersected repeat-masked regions and assembly gaps, and annotated all putative segmental duplications using the MAKER annotation results for supporting evidence. Candidate heterozygous deletions were defined as predicted deletion regions without windows, and a GC corrected read depth <5 [accounting for <6 and 16% of the total count of windows across scaled quail (v1.0) and bobwhite (v2.0), respectively]. RAPTR-SV was run using standard settings; however, split read alignment input was removed from the “cluster” step due to a higher number of split read alignments than expected. Subsequently, RAPTR-SV calls were based only on discordant read pairs. Overlap of RAPTR-SV tandem duplication predictions against the read-depth WSSD duplication predictions identified potential tandem duplicate scaffolds. Segmental duplications involving MAKER annotated genes were filtered for further investigation. To facilitate a comparative analysis, we used the CLC read mapper (v8.5.1) to reciprocally map genomic regions harboring predicted segmental duplications for the scaled quail and the bobwhite, thereby identifying both shared and unique segmental duplications predicted for both species (CLC Genomics Workbench 8.5.1, https://www.qiagenbioinformatics.com/). Putative repetitive regions were filtered, and the remaining tandem duplication candidates were manually examined in Interactive Genomics Viewer (Robinson *et al.* 2011). For technical quality control, we also extracted the sequences corresponding to the predicted bobwhite and scaled quail segmental duplications, and reciprocally aligned them to both draft genome assemblies, thereby providing the opportunity to assess whether putative orthologous regions were assembled and/or annotated for each species.

### Historic effective population size estimation

For comparison, a pairwise sequentially Markovian coalescent (PSMC) model was used to estimate the historic effective population size for the scaled quail and the bobwhite, as previously described (Li and Durbin 2011). Both PSMC input files were prepared according to the developers’ recommendations (Li and Durbin 2011). For the scaled quail (v1.0) genome, variants with ≤60× coverage and ≥350× coverage were filtered from the diploid consensus. Filtering cutoffs for variants within the bobwhite diploid consensus were ≤18× and ≥150×. The coefficient for downgrading mapping quality for reads with excessive mismatches was set at 50. Scaled quail (v1.0) and bobwhite (v2.0) scaffolds displaying blastn evidence to the sex chromosomes or mitochondrial genome of *T. guttata* or *G. gallus* were excluded. Estimated generation time for the scaled quail and the bobwhite was set at 1.22 yrs, as previously established for the bobwhite via survivorship studies (Halley *et al.* 2014). Moreover, for a general comparison with the bobwhite, and in the absence of any contradictory evidence, the per generation mutation rates of 1.1 × 10^−8^ and the PSMC default of 2.5 × 10^−8^ yr were used to calibrate sequence divergence to years, as previously described (Halley *et al.* 2014).

### Data availability

The whole genome shotgun project for the first version of the scaled quail genome has been deposited at DDBJ/EMBL//GenBank under the accession MCFN00000000, and the whole genome shotgun project for the second version of the bobwhite genome has been deposited at DDBJ/EMBL//GenBank under the accession AWGT00000000. Illumina sequence reads were also deposited in the SRA (accession numbers SRP068874 and SRP018482).

## Results and Discussion

### Sequencing and assembly

**Figure 1:**
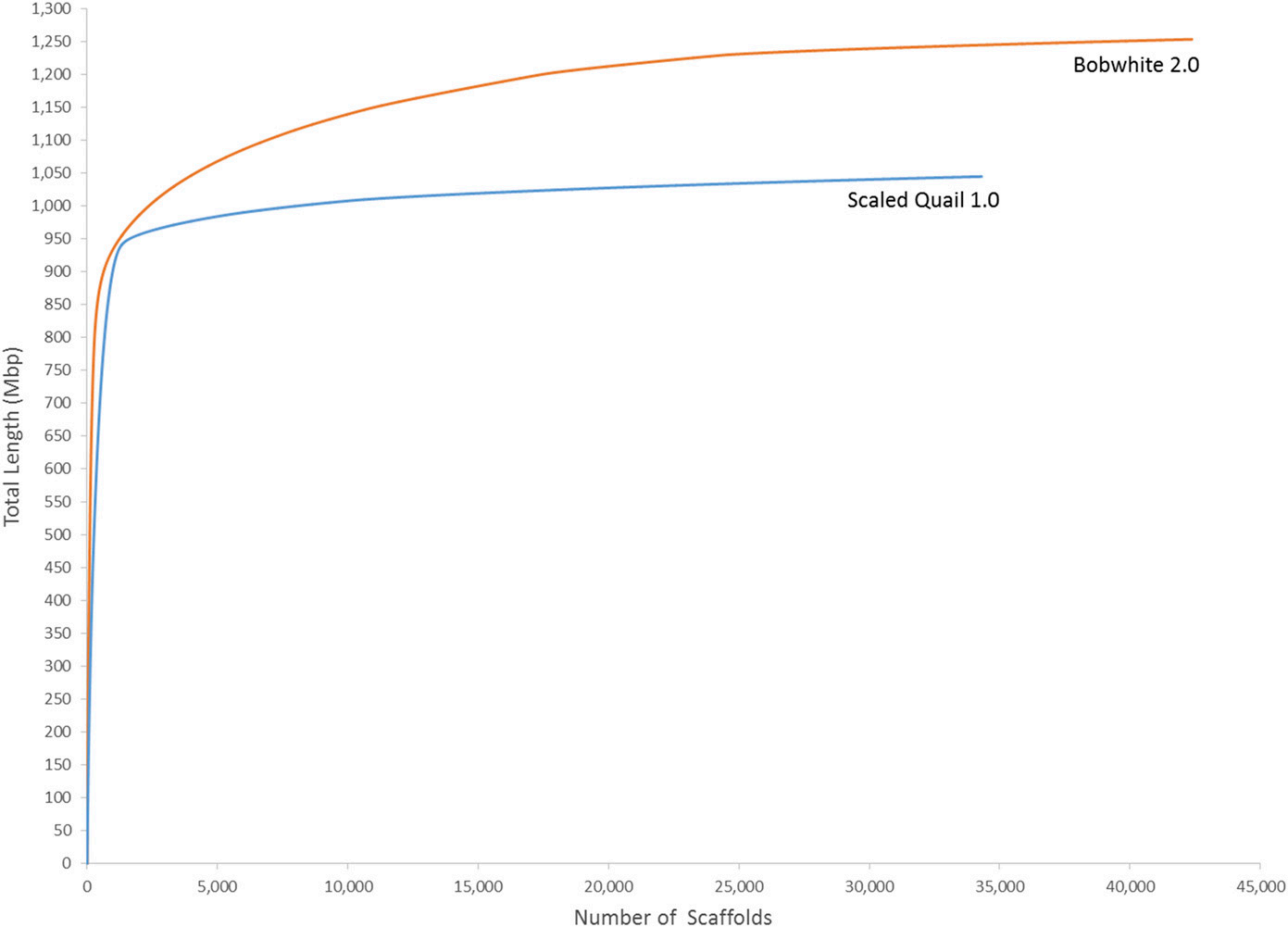
Scaled quail (*Callipepla squamata*) v1.0 and bobwhite (*Colinus virginianus*) v2.0 genome assemblies. Relationship between total scaffold length (megabase pair; Mbp) and total number of scaffolds.

Herein, we generated 532.81 and 300.41 Gb of raw Illumina sequence data for the scaled quail and the bobwhite, respectively. These data included reads from three small insert PE (295.98 Gb) and four MP (236.82 Gb) libraries for the scaled quail, and 1 PE (95.48 Gb) and 2 MP (204.92 Gb) libraries for the bobwhite. Using the k-mer based approach implemented in Jellyfish 2.0 (Marçais and Kingsford 2011), the estimated genome size for the scaled quail and the bobwhite was 1.08 and 1.23 Gb, respectively. These estimates are compatible with the first-generation bobwhite assembly (estimated 1.19–1.20 Gb; assembled 1.17 Gb) (Halley *et al.* 2014), assemblies produced for the chicken (assembled 1.05 Gb) as well as the turkey (assembled 1.075 Gb) (Hillier *et al.* 2004; Dalloul *et al.* 2010), and the general conservation of genome size established among birds (Tiersch and Wachtel 1991). The MaSuRCA assembly of paired reads for the scaled quail (v1.0) produced 34,302 final scaffolds (≥1 kb), with an N50 scaffold size of 1.035 Mb, whereas for the bobwhite, MaSurCA produced 42,369 scaffolds (≥1 kb) with an N50 scaffold size of 2.042 Mb. The weighted average of scaffold median coverage was 292× for the scaled quail, and 122× for the bobwhite. Collectively, the scaled quail assembly (v1.0) spanned 1.045 Gb, including 1.017 Gb of unambiguous sequence, and 0.028 Gb of gaps (N’s). The second-generation bobwhite assembly (v2.0) was ∼20% larger than that of the scaled quail, and spanned 1.254 Gb, which included 1.134 Gb of unambiguous sequence, and 0.120 Gb of gaps (N’s). Detailed summary data for the scaled quail (v1.0) and the bobwhite (v2.0) draft genome assemblies are provided in Table 1. Estimates of GC content for the assembled scaled quail and bobwhite genomes were similar (41.72% and 42.66%, respectively). Likewise, ≥90% of the assembled scaled quail and bobwhite genomes were captured within 1313 and 8990 scaffolds, respectively (Figure 1).

**Table 1:** Genome characteristics for the scaled quail (*Callipepla squamata*) v1.0, bobwhite (*Colinus virginianus*) v2.0, and bobwhite 1.1 (accession: AWGT01000000) genome assemblies

Genome Characteristics*^a^*	Scaled Quail 1.0	Bobwhite 2.0	Bobwhite 1.1*^b^*
Total scaffold length	1,045,281,893 bp	1,254,146,751 bp	1,095,702,334 bp
Scaffold length excluding gaps (N’s)	1,016,929,532 bp	1,134,249,133 bp	971,740,276 bp
Total scaffolds	34,302	42,369	65,748
N50 scaffold size	1,035,259 bp	2,042,136 bp	49,503 bp
Largest scaffold	4,990,493 bp	14,292,544 bp	600,658 bp
Average coverage	292×	122×	77×
Scaffolds capturing 90% total length	1313	8990	25,837

a Scaffolds shorter than 1 kb are excluded from the assemblies.

b First-generation scaffolded bobwhite assembly (Halley *et al.* 2014).

A justified skepticism of measuring *de novo* genome assembly quality based on fragment length distributions exists, which can be addressed using orthologous gene sets built from species with well-established reference genomes (Parra *et al.* 2009; Waterhouse *et al.* 2013; Simão *et al.* 2015). Gene space coverage assessments based on benchmarking sets of universal single-copy orthologs have proven successful measures of both genome assembly and annotation quality (as implemented within the program BUSCO) (Waterhouse *et al.* 2013; Simão *et al.* 2015). BUSCO analysis was used for assembly validation and quality assessment of the scaled quail (v1.0) and bobwhite (v2.0) draft genome assemblies, and subsequently compared to other well-established avian genome assemblies. Of the 3023 single-copy orthologs searched (Simão *et al.* 2015), 84.8% were detected as complete and single-copy in the scaled quail genome (v1.0), whereas 82.5% were detected as complete and single copy for the bobwhite (v2.0). In comparison, BUSCO reported 85.8% for the chicken genome (*G. gallus* 5.0), 76.1% for the zebra finch genome (*T. guttata* 3.2.4), and 74.1% for the turkey genome (*M. gallopavo* 5.0). Moreover, comparative BUSCO analyses involving 21 additional high-coverage avian genomes (Huang *et al.* 2013; Kawahara-Miki *et al.* 2013; Shapiro *et al.* 2013; Zhang *et al.* 2014) revealed further evidence for a very high level of completeness associated with the draft scaled quail (v1.0) and bobwhite (v2.0) genome assemblies. Nevertheless, genomes assembled to the level of chromosomes will always be more useful than those still at the scaffold stage. Detailed summary and comparative data for all BUSCO analyses are provided in Table 2 and in Supplemental Material, File S1.

**Table 2:** BUSCO single-copy vertebrate ortholog detection summary for the scaled quail (*Callipepla squamata*) v1.0, bobwhite (*Colinus virginianus*) v2.0, chicken (*Gallus gallus*) 4.0, chicken 5.0, zebra finch (*Taeniopygia guttata*) 3.2.4, and turkey (*Meleagris gallopavo*) 5.0 genome assemblies

	Scaled Quail 1.0	Bobwhite 2.0	Chicken 4.0	Chicken 5.0	Zebra Finch 3.2.4	Turkey 5.0
Complete single-copy BUSCOs	2565 (84.8%)	2494 (82.5%)	2547 (84.3%)	2593 (85.8%)	2299 (76.1%)	2241 (74.1%)
Complete duplicated BUSCOs	45 (1.5%)	48 (1.6%)	30 (1.0%)	62 (2.1%)	73 (2.4%)	18 (0.6%)
Fragmented BUSCOs	156 (5.2%)	176 (5.8%)	178 (5.9%)	155 (5.1%)	244 (8.1%)	303 (10.0%)
Missing BUSCOs	257 (8.5%)	305 (10.1%)	268 (8.9%)	213 (7.0%)	407 (13.5%)	461 (15.2%)
Total BUSCO groups searched	3023	3023	3023	3023	3023	3023

### Repetitive content and variant prediction

The minimum estimated repetitive DNA content for the scaled quail (v1.0) and bobwhite (v2.0) draft genomes was 10.40% and 10.43%, respectively (Table 3). These estimates are lower than similar RepeatMasker predictions for the chicken (16.13%, *G. gallus* 5.0), and higher than those for the zebra finch (7.83%, *T. guttata* 3.2.4), turkey (8.22%, *M. gallopavo*, UMD_5.0), and scarlet macaw (4.00%, *Ara macao*, SMACv1.1) genome assemblies (Smit *et al.* 1996; Warren *et al.* 2010, 2017; Seabury *et al.* 2013; Dalloul *et al.* 2014). In comparison to the first-generation bobwhite genome assembly, where the minimum repetitive content was estimated at 8.08% (Halley *et al.* 2014), longer reads utilized here coupled with advanced assembly and scaffolding algorithms produced a bobwhite genome assembly that was ∼8% larger (1.047 Gb *vs.* 1.134 Gb of unambiguous sequence), thereby resolving the complexity of previously undetected repetitive content (Pop 2009; Treangen and Salzberg 2012; Halley *et al.* 2014). The high proportion of LINES predicted in both quail assemblies (Table 3) is also consistent with previous analyses of multiple avian genomes, including but not limited to, the first-generation bobwhite, scarlet macaw, chicken, zebra finch, turkey, peregrine falcon, saker falcon, and golden eagle genome assemblies (Hillier *et al.* 2004; Dalloul *et al.* 2010; Warren *et al.* 2010; Seabury *et al.* 2013; Zhan *et al.* 2013; Doyle *et al.* 2014; Halley *et al.* 2014). A comparative summary of repetitive content predicted within the scaled quail (v1.0) and bobwhite (v2.0) genome assemblies is depicted in Table 3.

**Table 3:** Repetitive content summary for the scaled quail (*Callipepla squamata*) v1.0 and northern bobwhite (*Colinus virginianus*) v2.0 genomes

Repeat Type Predicted	Scaled Quail v1.0		Bobwhite v2.0	
	Total Elements	Total bp (% of Genome)	Total Elements	Total bp (% of Genome)
SINEs	4847	622,077 (0.06)	4819	617,239 (0.05)
LINEs	179,965	58,033,984 (5.55)	182,570	57,752,214 (4.60)
LTR elements	42,752	18,043,711 (1.73)	40,895	16,316,942 (1.30)
DNA transposons	30,287	9,285,701 (0.89)	30,754	9,302,514 (0.74)
Unclassified interspersed repeats	2715	448,390 (0.04)	2675	446,377 (0.04)
Small RNA	2417	273,356 (0.03)	2284	266,713 (0.02)
Satellites	14,908	3,811,752 (0.36)	11,211	3,430,821 (0.27)
Simple repeats	310,838	14,852,766 (1.42)	522,056	38,021,885 (3.03)
Low complexity	56,975	3,349,638 (0.32)	69,601	4,715,268 (0.38)
Total	645,704	108,721,375 (10.40)	866,865	130,869,973 (10.43)

Despite reductions in cost associated with genome sequencing and/or single nucleotide polymorphism (SNP) genotyping, microsatellites have continued to retain much of their utility as markers for population genetic studies (Jarne and Lagoda 1996; Schlotterer 2000; Li *et al.* 2002; Schable *et al.* 2004; Faircloth *et al.* 2009; Berkman *et al.* 2013a,b; Orange *et al.* 2014), particularly among wildlife species where funding or research tools are limited. Likewise, simple sequence repeats such as microsatellites within coding and noncoding regions have been proposed to modulate both regulatory effects and qualitative protein variation that has been associated with morphological evolution and adaptation (Fondon and Garner 2004; Kashi and King 2006). Therefore, we provide a comprehensive assessment of microsatellites within the scaled quail (v1.0) and bobwhite (v2.0) genomes. For the scaled quail, we predicted 2,785,005 microsatellites using PHOBOS, including 372,187 di-, 510,682 tri-, 521,803 tetra-, 570,266 penta-, 493,694 hexa-, 151,525 hepta-, 104,165 octa-, 32,589 nona-, and 28,094 deca-nucleotide tandem repeats. An equivalent search of the bobwhite genome (v2.0) produced evidence for 3,819,225 microsatellites, including 686,315 di-, 971,826 tri-, 625,888 tetra-, 580,335 penta-, 605,509 hexa-, 161,335 hepta-, 112,744 octa-, 41,737 nona-, and 33,536 deca-nucleotide tandem repeats. Notably, for the scaled quail we detected ∼1 million fewer tandem repeats than for the bobwhite, which was mostly accounted for by a lack of di- and trinucleotide motifs. The average density of one microsatellite per 365 kb observed for the scaled quail genome (v1.0; excluding N’s) is more consistent with the previously proposed dispersion of microsatellites (1/382 kb) within avian genomes (Neff and Gross 2001) than the average density observed for the bobwhite genome (1/297 kb; v2.0; excluding N’s). To date, >50 unique microsatellite loci have been characterized for the scaled quail and the bobwhite, collectively, and some have been utilized for population studies (Schable *et al.* 2004; Faircloth *et al.* 2009; Berkman *et al.* 2013a,b; Orange *et al.* 2014). However, the generation of high-quality draft genome assemblies with precise coordinates for millions of genome-wide microsatellites may prove useful for the selection of neutral or nearly neutral markers that would facilitate future low-cost population analyses, and/or the investigation of hybridization. To further enable such analyses, we used blastn to identify the scaffold locations for all of the previously reported microsatellites (Schable *et al.* 2004; Faircloth *et al.* 2009; Orange *et al.* 2014) within the bobwhite (v2.0) and scaled quail (v1.0) genome assemblies; with all microsatellites independently detected by PHOBOS (see File S1). Moreover, we also performed a reciprocal search that sought to determine whether some microsatellites might be useful in both species, and found evidence for 23 shared microsatellites (see File S1). While natural and experimental hybridization has been documented between the bobwhite and the scaled quail (Johnsgard 1970; Shupe 1990), the extent of historic hybridization has not been estimated using nuclear, genome-wide variation. However, a recent mitochondrial population study for the bobwhite (*n* = 53), which also produced a complete scaled quail mitochondrial genome for comparison (GenBank accession KT722338), failed to detect any evidence of historic hybridization (bobwhite ♂ × scaled quail ♀), despite sampling from regions where both species naturally occur (Halley *et al.* 2015). A subset of the nuclear microsatellites presented here may prove useful to facilitate low-cost investigations of management concerns using larger sample sizes, including issues related to population structure, gene flow, and nuclear introgression between the scaled quail and bobwhite.

Beyond microsatellite loci, we also predicted genome-wide autosomal sequence variation resulting from biparental inheritance of alternative alleles (heterozygosity) for the scaled quail (v1.0) and the bobwhite (v2.0). In the first characterization of putative genome-wide sequence variation for the scaled quail, we detected 1,852,113 SNVs, 41,048 MNVs, 193,971 small indels, and 7435 small nucleotide replacements (*i.e.*, AAA→CC), thereby yielding an overall autosomal variant density of ∼2.48 variants per kilobase. In striking contrast, we detected 3,444,693 SNVs, 85,350 MNVs, 348,817 small indels, and 12,554 small replacements for the bobwhite, which revealed an overall autosomal variant density of ∼4.17 variants per kilobase. This is ∼29.5% higher than the 3.22 autosomal variants per kilobase previously reported for the first-generation bobwhite draft genome assembly (Halley *et al.* 2014). Differences in average autosomal variant density described here for the second-generation bobwhite draft genome assembly (v2.0) can be attributed to the generation of a larger and more complete draft genome assembly (1.254 Gbp *vs.* 1.096 Gbp), as well as the inclusion of MNVs and small nucleotide replacements for the reported density estimates.

### Annotation and quality control analyses

The scaled quail (v1.0) and bobwhite (v2.0) draft genome assemblies were annotated using the automated MAKER pipeline, with an emphasis on high-accuracy prediction of protein coding genes, using only evidence from existing avian protein and cDNA sets (Cantarel *et al.* 2008; Campbell *et al.* 2014). This allowed for annotations of the scaled quail and bobwhite genomes in the absence of large-scale annotation teams, and without prior species specific protein coding sequence data for the scaled quail.

**Figure 2:**
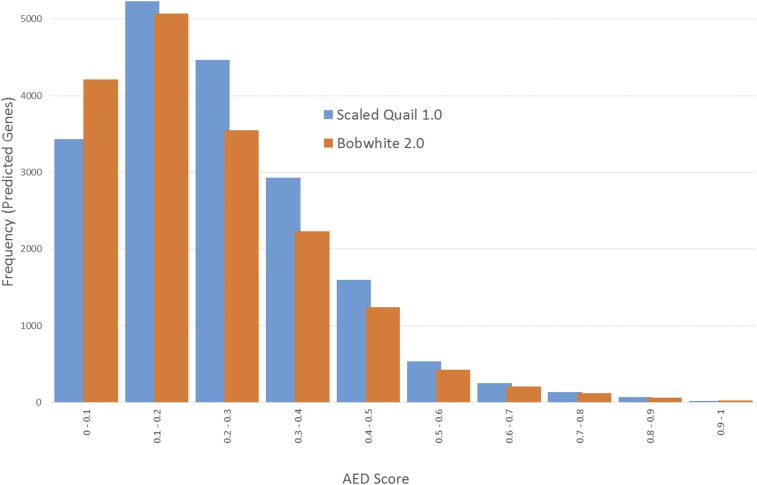
Scaled quail (*Callipepla squamata*) v1.0 and Bobwhite (*Colinus virginianus*) v2.0 gene AED scores. Bobwhite scores are shown in orange; scaled quail scores are shown in blue.

**Figure 3:**
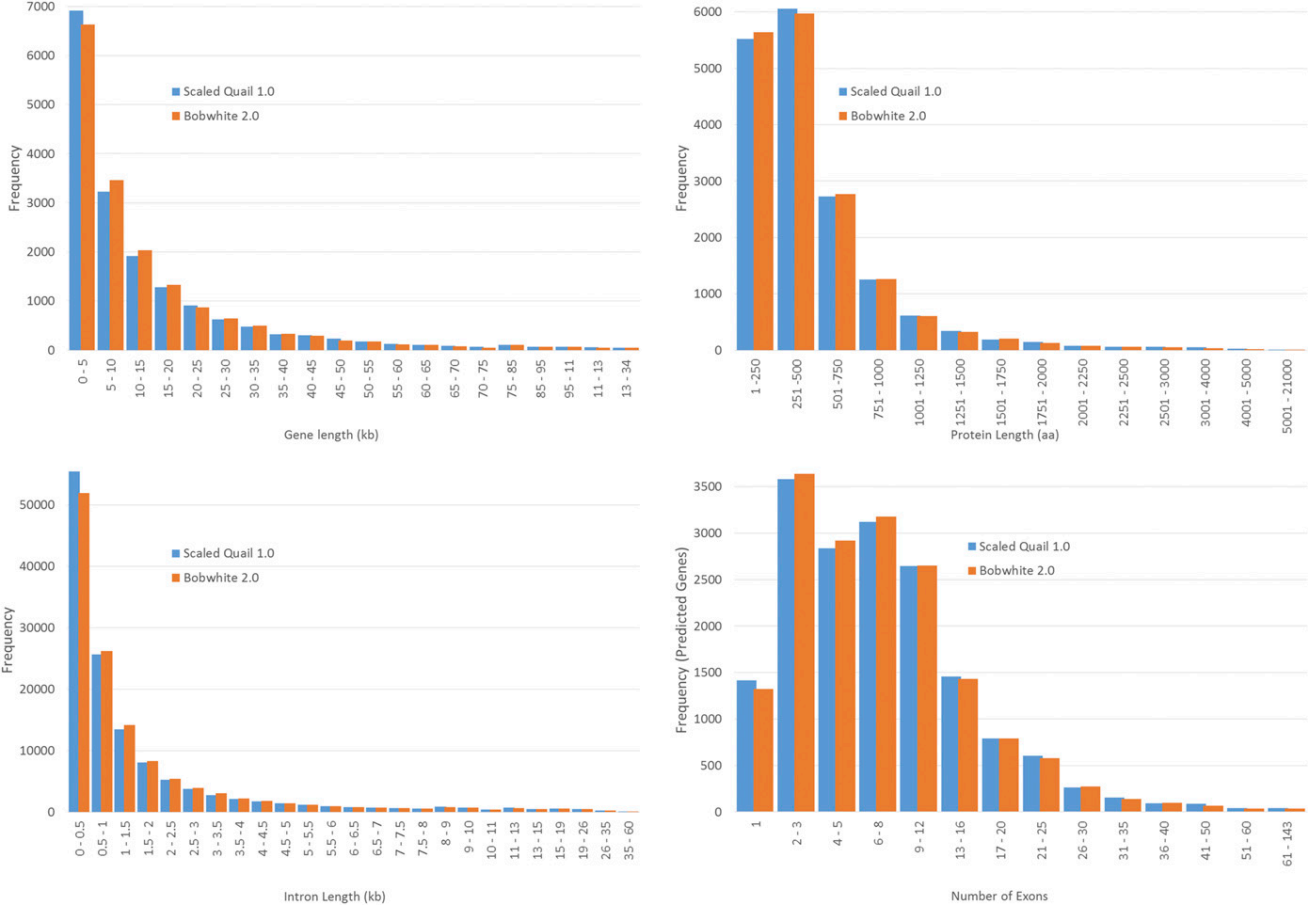
Scaled quail (*Callipepla squamata*) v1.0 and bobwhite (*Colinus virginianus*) v2.0 MAKER annotation summary frequency distributions. Units expressed as kilobase (kb) and amino acids (aa).

The putative protein-coding gene set for the scaled quail (v1.0) consisted of 17,131 genes, as predicted *in silico* using the MAKER pipeline with evidence from chicken, turkey, zebra finch, and the bobwhite. Of the MAKER predicted genes, 95% have Annotation Edit Distance (AED) scores ≤0.5, and 20% have AED scores ≤0.1, thereby suggesting a reliable protein-coding gene set, despite the absence of species-specific cDNA evidence (Campbell *et al.* 2014) (Figure 2). The average exon length for the final scaled quail gene set was 173 bp, which is compatible with previous data related to bird exon lengths (Hughes and Hughes 1995). The average intron length was 1695 bp (Figure 3), which is in agreement with more recent findings on intron sizes for the chicken, turkey, and zebra finch (Zhang and Edwards 2012). A blastp search against the UniProt Swiss-Prot database via the MAKER protocol resulted in 15,711 (92%) predicted homologies (Camacho *et al.* 2009; Campbell *et al.* 2014; The UniProt Consortium 2015).

Annotation analyses of the bobwhite (v2.0) draft genome assembly using the MAKER pipeline produced a putative protein coding gene set consisting of 17,165 genes, with a mean exon length of 174 bp, and mean intron length of 1692 bp. Similar to the scaled quail, the distribution of AED scores for the bobwhite were skewed toward smaller distance values, with 95% producing scores ≤0.5 and 25% producing scores ≤0.1 (Figure 2 and Figure 3). Again, a blastp search against the UniProt Swiss-Prot database was used and predicted homologies were assigned for 15,824 (92%) genes (Camacho *et al.* 2009; Campbell *et al.* 2014; The UniProt Consortium 2015). While we recognize the limitations of *ab initio* gene prediction that rely heavily on alternate species’ evidence (Seabury *et al.* 2013; Halley *et al.* 2014), the annotation sets produced in this study provide a strong foundation for future scaled quail and bobwhite genomics research.

Quality control analyses aimed at identifying nonavian scaffolds via blast (blastn, blastx), and the evaluation of read depth, led to the removal of one scaffold from the scaled quail assembly (v1.0), and two scaffolds from the bobwhite assembly (v2.0); based on convincing evidence that these scaffolds were not produced from the targeted avian genomes. The scaffold removed from the scaled quail assembly was 1287 bp in length, and produced a blastn alignment with 100% identity (across 1287 bp) to the bacterium *Dietzia timorensis* (strain ID05-A0528) complete genome (accession: CP015961.1), indicating the presence of an unknown bacterium, and produced no meaningful blastn alignments to the chicken or zebra finch genomes (MaSuRCA scaffold ID: jcf7180005140066; File S2). The two scaffolds excluded from the bobwhite (v2.0) genome assembly were previously reported to produce convincing blastn and blastp alignments to apicomplexan parasite sequences in the bobwhite (v1.0) assembly (Halley *et al.* 2014; Orosz 2015). The MaSuRCA scaffolds jcf7180006226006 and jcf7180006136291 were independently assembled updates to contig 108821 (AWGU01108821) and contig 45101 (AWGU01045101) in the first-generation bobwhite assembly, and are included in File S2. Scaffold jcf7180006136291 produced a blastn alignment with 76% identity to the *Toxoplasma gondii* apicoplast complete genome (accession U87145.2) across 99% of the scaffold (8761 bp), and could represent a portion of an apicoplast sequence. Scaffold jcf7180006226006 produced a blastn alignment with 72% identity to *Sarcocystis neurona* (strain SN3, clone E1, contig 00498) whole genome shotgun sequence (accession: JAQE01000498.1) across 37% of the scaffold length (357,155 bp). The query coverage of 37% is the result of 731 high scoring segment pairs (HSPs), the longest of which covers 5870 bp of the scaffold sequence (1.6%). We removed this scaffold from the assembly under the suspicion that it may potentially have nonavian origin(s). It should also be noted that the majority of the alignments reported by Orosz (2015) within blast summary tables are actually very short alignments, regardless of contig or scaffold size, with most being insufficient evidence for the reported inferences. Specifically, of the 29 unique bobwhite sequences reported by Orosz (2015) as putative “contamination,” 19 alignments covered <6% of the bobwhite query sequence (and 15 covered <1%), with bobwhite contig length ranging from 2988 to 70,570 bp. The top HSP alignment lengths ranged from 30 to 174 bp for these 19 alignments, and nine of 19 alignments were <50 bp long. This raises the possibility for spurious assignment of these sequences as contaminating DNA from host parasites (Pertsemlidis and Fondon 2001).

### Copy number variant (CNV) analysis

CNV studies in birds have several technical advantages over those in mammals, which stems primarily from having smaller genome sizes and lower numbers of interspersed repeats, segmental duplications, and pseudogenes (Hillier *et al.* 2004; Völker *et al.* 2010). CNVs in avian genomes have been hypothesized to be driving forces for evolution and speciation (Coghlan *et al.* 2005; Griffin *et al.* 2008; Völker *et al.* 2010). Herein, we detected 256 putative segmental duplications in the scaled quail genome (v1.0) and 711 in the bobwhite genome (v2.0) based on WSSD. Reciprocal alignments via blastn were performed to further validate the disparity in total predicted segmental duplications (*i.e.*, 256 *vs.* 711) by searching for each associated sequence in both genomes, thereby yielding overwhelming support for a biological origin (see File S3). Among these, 60 scaled quail and 147 bobwhite putative segmental duplications intersect with 53 and 113 unique, annotated genes, respectively, within their respective MAKER annotation sets (File S3).

Notable findings included putative segmental duplications intersecting genes with homology to olfactory receptor, mucin, feather keratin, and centromere protein C (*CENP-C*) genes (File S3). Segmental duplication of olfactory receptor-like gene families is well characterized in vertebrate genomes (Niimura and Nei 2005; Vandewege *et al.* 2016), and recent findings indicate that avian olfaction is more developed than previously thought (Hagelin 2006; Amo *et al.* 2008; Steiger *et al.* 2009). However, the two quail olfactory receptor-like genes detected as putative segmental duplications are possibly nonfunctional pseudogenes due to their lack of transmembrane domains; with only four and six of the seven typical olfactory receptor transmembrane domains detected (MAKER IDs: *ASZ278_16298*; *ASZ78_16340*) using the Simple Modular Architecture Research Tool (SMART) (Schultz *et al.* 1998; Letunic *et al.* 2015), thereby violating previously established criteria for filtering possible functional olfactory receptors (Niimura and Nei 2003). *AXZ278_16298* also appears shorter than the length cut-off previously established for functional transmembrane domains (Niimura and Nei 2003). It was somewhat surprising, however, that 121 MAKER predicted genes in the scaled quail annotation set have homology to olfactory receptors, as compared to only 56 in the bobwhite annotation set, possibly reflecting a functional expansion of olfactory receptors in the scaled quail lineage. However, this result may also be influenced by repetitive and/or difficult to assemble genomic regions close to olfactory receptor genes, thus creating unbalanced detection among the two assemblies (scaffolds ≥1 kb). The number of potential olfactory receptor gene homologs found in our annotation sets are lower than the 479 and 553 olfactory receptor gene homologs found in the chicken and zebra finch genomes, respectively (Steiger *et al.* 2009). However, it should be noted that our methodology strictly relied on a search of MAKER annotated genes with predicted homology rather than a direct genome-wide blast search for homology to these genes (Steiger *et al.* 2009). A shared putative segmental duplication in the scaled quail (v1.0) and bobwhite (v2.0) draft genomes overlaps with two MAKER predicted genes (MAKER ID: *ASZ78_12344* in the scaled quail; *H355_12445* in the bobwhite) that have homology with the human mucin gene *MUC16*. Notably, tandem clusters of mucin gene families have previously been noted in the human genome, and *MUC16*, which is an epithelial glycoprotein known to modulate inhibitory interactions with natural killer cells, has also been associated with human tumor cell growth (Patankar *et al.* 2005; Tuzun *et al.* 2005; Thériault *et al.* 2011). Keratin gene duplications are also well described in avian genomes (Gregg *et al.* 1984; Greenwold and Sawyer 2010), and it is therefore relatively unsurprising that our analyses provided evidence of two putative segmental duplications for the bobwhite that overlap with feather keratin-like genes (MAKER IDs: *H355_16813* and *H355_14481* in the bobwhite). Duplicated regions of *CENP-C* are also known to exist in several animal and plant species (Talbert *et al.* 2004); however, a putative segmental duplication predicted for the bobwhite *CENP-C* homolog (MAKER ID: *H355_00001* in the bobwhite) intersects with only three of 13 predicted exons. Notably, individual *CENP-C* exon duplications and deletions have been detected and determined to be adaptive among grass species (Talbert *et al.* 2004). Likewise, several studies have reported evidence for adaptive evolution operating on *CENP-C* in plants and mammals (Talbert *et al.* 2004; Schueler *et al.* 2010).

Reciprocal mapping of segmental duplications predicted for the bobwhite onto those predicted for the scaled quail revealed 77 putative segmental duplications that were predicted to be shared within both draft genomes, which is suggestive of the ancestral state. Collectively, 28 of these putative segmental duplications were determined to intersect the MAKER predicted scaled quail gene set. However, only 15 of the 28 genes in each species had similar predicted homologies (File S4). Our analyses confirmed shared predicted segmental duplications with homology to genes encoding olfactory receptor 14J1, mucin-16, Shroom3, septin-11, and RanBP2 (File S4). RanBP2 is a nuclear membrane protein involved in the nuclear pore complex (Sakin *et al.* 2015). Both septin-11 and Shroom3 play roles in cell process and are involved in cell membrane shape (Hall and Russell 2012; Yeo *et al.* 2015). However, because segmental duplications exist as interspersed or tandem low copy repeats, we conducted additional analyses based on read eversions to identify candidate regions of putative tandem duplication. Strong evidence for tandem duplications within regions of the scaled quail genome assembly (v1.0) was noted for three scaffolds (jcf7180005234811, jcf7180005198788, and jcf7180005234681), the first of which reciprocally maps to a putative segmental duplication detected in the bobwhite genome (v2.0) that overlaps with a gene that has homology to *SHROOM3* (MAKER ID: *ASZ78_15687* in the scaled quail; *H355_16938* in the bobwhite). Putative heterozygous deletions were also predicted by our WSSD read depth analysis, thereby revealing 13,165 and 4104 regions of low read depth in the scaled quail (v1.0) and bobwhite (v2.0) genome assemblies, respectively. These regions are likely to represent heterozygous interchromosomal deletions due to biparental inheritance of alternative alleles; however, we cannot rule out the possibility that these regions are fine-scale misassemblies that evaded our read depth detection method. Future population analyses using WSSD for the scaled quail and the bobwhite are expected to yield further insight regarding the genomic landscape of both ancestral and derived copy number variants.

### Comparison of historic effective population size (*N*_e_)

**Figure 4:**
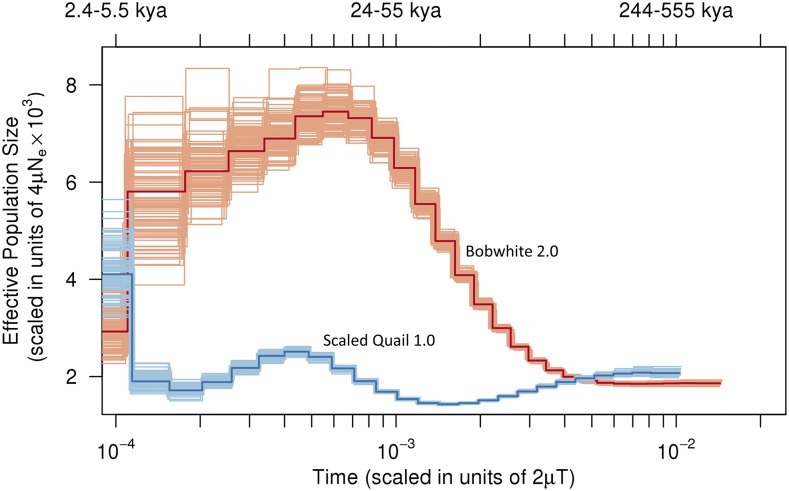
Comparative demographic history analysis and PSMC effective population size estimates for the scaled quail (*Callipepla squamata*) v1.0 (blue) and the bobwhite (*Colinus virginianus*) v2.0 (orange). Historic estimates of effective population size are presented on the *y*-axis as the scaled mutation rate. The lower *x*-axis represents pairwise sequence divergence (per-site), and the upper *x*-axis represents years before present, both on a log scale. Generation intervals of 1.22 yr were used for the scaled quail and the bobwhite (See *Materials and Methods*). Given the absence of any known per-generation *de novo* mutation rates for the scaled quail and the bobwhite, we used the two human mutation rates (μ) of 1.1 × 10^−8^ and 2.5 × 10^−8^ per generation (see *Materials and Methods*). Darker lines represent population size inferences, while lighter, thinner lines represent 100 bootstraps to quantify the uncertainty of the inferences.

Using high-quality autosomal SNV data in conjunction with a PSMC model (Li and Durbin 2011), we reconstructed the demographic histories of the scaled quail (v1.0) and the bobwhite (v2.0). Given the broad geographic range of the bobwhite, with the potential for a large theoretical census size, as compared to the restricted range and much smaller theoretical census size for the scaled quail, we hypothesized that historic *N*_e_ for the bobwhite would be larger. Notably, larger census sizes are also expected to produce more opportunities for both mutation and recombination. Therefore, it is relatively unsurprising that estimates of historic *N*_e_ for the scaled quail were uniformly lower than those estimated for the bobwhite across all time points in our coalescent models (Figure 4 and File S5). Moreover, peak *N*_e_ estimated for the bobwhite was about three times larger than that of the scaled quail. A notable decline in bobwhite estimated *N*_e_ was detected beginning ∼20 KYA, which is coincident with the timing of modern human colonization of the New World (Eshleman *et al.* 2003; Gilbert *et al.* 2008; Waters *et al.* 2011a,b), the collapse of the megafauna (Alroy 2001; Pushkina and Raia 2008), and the last glacial maximum (LGM) (Yokoyama *et al.* 2000; Clark and Mix 2002), as previously detected and described using the first-generation bobwhite draft genome assembly, and shorter reads (Halley *et al.* 2014). Similar precipitous declines in estimated *N*_e_ detected for the scaled quail appear to be younger, beginning ∼15 KYA, and may be related to a historic range that was largely restricted to southern grasslands, thereby delaying glacial declines via temporary refugia. However, it should be noted that measures of modern diversity resulting from biparental inheritance of alternative alleles (*i.e.*, heterozygosity) were much lower for the scaled quail (v1.0), as compared with the bobwhite. While diversity at neutral or nearly neutral loci may not always be predictive of adaptability (Yonekura *et al.* 2007), population fitness is significantly correlated with heterozygosity, and therefore, the scaled quail may potentially be more sensitive to biological phenomena that result in reduced heterozygosity (*i.e.*, bottlenecks, habitat fragmentation with inbreeding, etc.) (Reed and Frankham 2003; Markert *et al.* 2010). Moreover, while this inference is not intended to prioritize the conservation and/or management of the scaled quail in relation to the bobwhite, it is important to establish baseline estimates of genome-wide diversity (*i.e.*, heterozygosity) for any species in decline. Draft genome assemblies and corresponding estimates of modern genome-wide diversity reported here are expected to enable future population comparisons as well as genetic monitoring within and between natural bobwhite and scaled quail populations.

Divergence times between the scaled quail and the bobwhite have previously been estimated at 8.80 MYA (median; range = 6.38–11.23 MYA), with estimates based on nuclear genes and the mitochondria (http://www.timetree.org) (Hedges *et al.* 2006; Jetz *et al.* 2012). While the range of divergence times between avian species that produce fertile hybrids is relatively large, an average divergence time of 7 MYA and 14% mitochondrial nucleotide divergence has been indicated in complete hybrid infertility (Price 2008). Notably, only 7% mitochondrial nucleotide divergence exists between the scaled quail and the bobwhite (Halley *et al.* 2015), which likely reflects a more recent split from a common ancestor. Future population genomics studies are expected to shed additional light on historic hybridization events between the scaled quail and the bobwhite.

### Conclusions

Herein we have assembled and annotated a first-generation (v1.0) draft genome for the scaled quail, and a second-generation (v2.0) draft genome for the bobwhite. Analyses that use a conserved set of nuclear orthologs to assess genome completeness indicated that the scaled quail (v1.0) and bobwhite (v2.0) draft genomes are comparative in their completeness to other more established avian genomes (*i.e.*, chicken, turkey, zebra finch, and 23 additional avian genomes; see File S1). Modern estimates of genome diversity (*i.e.*, heterozygosity) for the bobwhite were >1.5 times higher than those predicted for the scaled quail, which also resulted in the estimation of a much larger historic effective population size for the bobwhite, and both species were predicted to experience precipitous declines beginning ∼15–20 KYA. The genome resources reported here also facilitated the first sequence-based analysis of copy number variants for the scaled quail and the bobwhite, which provided evidence of both shared (*i.e.*, ancestral) and derived segmental duplications within the scaled quail and bobwhite draft genomes. The genome resources described here provide a solid foundation and natural segue to population and comparative genomics studies for these species.

## Supplementary Material

Supplemental material is available online at www.g3journal.org/lookup/suppl/doi:10.1534/g3.117.043083/-/DC1.
