# Supplementary material for: Annotated Draft Genome Assemblies for the Northern Bobwhite (Colinus virginianus) and the Scaled Quail (Callipepla squamata) Reveal Disparate Estimates of Modern Genome Diversity and Historic Effective Population Size
Source: G3 (Bethesda). 2017 Jul 17;7(9):3047–58. doi: 10.1534/g3.117.043083 (PMC5592930; doi:10.1534/g3.117.043083)
Supplement: Supplementary file 4 [file 3047FileS4.docx]

**Table S4 Reciprocal Gene Homology For Predicted Segmental Duplications: MAKER predicted gene IDs in the scaled quail (*Callipepla squamata*) v1.0 and bobwhite (*Colinus virginianus*) v2.0 annotations with predicted UniProt Swiss-Prot Homology**

| **Scaled Quail v1.0** | | **Northern Bobwhite v1.0** | |
| --- | --- | --- | --- |
| **Gene ID** | **UniProt Swiss-Prot Homology** | **Gene ID** | **UniProt Swiss-Prot Homology** |
| *ASZ78_15687* | *SHROOM3: Protein Shroom3 (Homo sapiens)* | *H355_16938* | *SHROOM3: Protein Shroom3 (Homo sapiens)* |
| *ASZ78_12344* | *MUC16: Mucin-16 (Homo sapiens)* | *H355_12445* | *MUC16: Mucin-16 (Homo sapiens)* |
| *ASZ78_16329* | *RGPD4: RanBP2-like and GRIP domain-containing protein 4 (Homo sapiens)* | *H355_04479* | *Ranbp2: E3 SUMO-protein ligase RanBP2 (Mus musculus)* |
| *ASZ78_16298* | *OR14J1: Olfactory receptor 14J1 (Homo sapiens)* | *H355_10492* | *OR14J1: Olfactory receptor 14J1 (Homo sapiens)* |
| *ASZ78_16250* | *Phf7: PHD finger protein 7 (Mus musculus)* | *H355_12648* | *Similar to PHF7: PHD finger protein 7 (Homo sapiens)* |
| *ASZ78_08328* | *Protein of unknown function* | *H355_07796* | *Protein of unknown function* |
| *ASZ78_16529* | *G2e3: G2/M phase-specific E3 ubiquitin-protein ligase (Mus musculus)* | *H355_00914* | *G2e3: G2/M phase-specific E3 ubiquitin-protein ligase (Mus musculus)* |
| *ASZ78_16491* | *GTSF1: Gametocyte-specific factor 1 (Macaca fascicularis)* | *H355_15932* | *GTSF1: Gametocyte-specific factor 1 (Macaca fascicularis)* |
| *ASZ78_15713* | *SEPT11: Septin-11 (Homo sapiens)* | *H355_00921* | *SEPT11: Septin-11 (Homo sapiens)* |
| *ASZ78_15826* | *Mroh7: Maestro heat-like repeat-containing protein family member 7 (Mus musculus)* | *H355_01541* | *MROH5: Maestro heat-like repeat family member 5 (Homo sapiens)* |
| *ASZ78_16340* | *OR14J1: Olfactory receptor 14J1 (Homo sapiens)* | *H355_16939* | *OR14J1: Olfactory receptor 14J1 (Homo sapiens)* |
| *ASZ78_16235* | *Ccdc81: Coiled-coil domain-containing protein 81 (Rattus norvegicus)* | *H355_16607* | *Ccdc81: Coiled-coil domain-containing protein 81 (Rattus norvegicus)* |
| *ASZ78_13406* | *RANBP2: E3 SUMO-protein ligase RanBP2 (Pan troglodytes)* | *H355_04479* | *Ranbp2: E3 SUMO-protein ligase RanBP2 (Mus musculus)* |
| *ASZ78_14811* | *Shroom3: Protein Shroom3 (Mus musculus)* | *H355_00917* | *SHROOM3: Protein Shroom3 (Homo sapiens)* |
| *ASZ78_16561* | *Ranbp2: E3 SUMO-protein ligase RanBP2 (Mus musculus)* | *H355_16900* | *Ranbp2: E3 SUMO-protein ligase RanBP2 (Mus musculus)* |
