# Supplementary material for: Annotated Draft Genome Assemblies for the Northern Bobwhite (Colinus virginianus) and the Scaled Quail (Callipepla squamata) Reveal Disparate Estimates of Modern Genome Diversity and Historic Effective Population Size
Source: G3 (Bethesda). 2017 Jul 17;7(9):3047–58. doi: 10.1534/g3.117.043083 (PMC5592930; doi:10.1534/g3.117.043083)
Supplement: Supplementary file 5 [file 3047FileS5.docx]

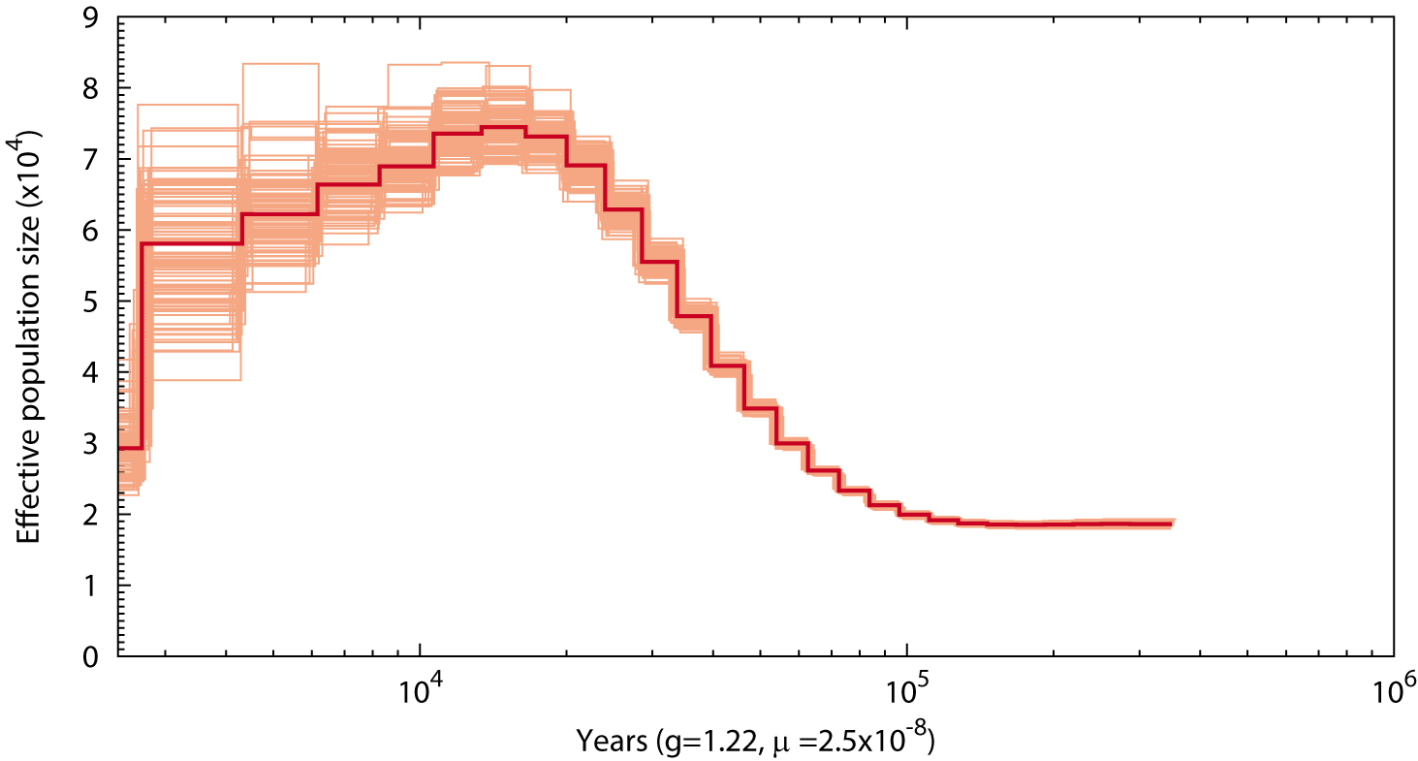


**S5 Demographic History Analysis and PSMC Effective Population Size Estimates for the Bobwhite (*Colinus virginianus*) v2.0.** Historic estimates of effective population size are presented on the y-axis. The x-axis represents years before present, on a log scale. Generation intervals of 1.22 years were used for the bobwhite (See Methods). Given the absence of any known per-generation *de novo* mutation rates for the scaled quail and the bobwhite, we used the two human mutation rates (μ) of 1.1×10−8 and 2.5×10−8 per generation (See Methods). Darker lines represent population size inferences, while lighter, thinner lines represent 100 bootstraps to quantify the uncertainty of the inferences.


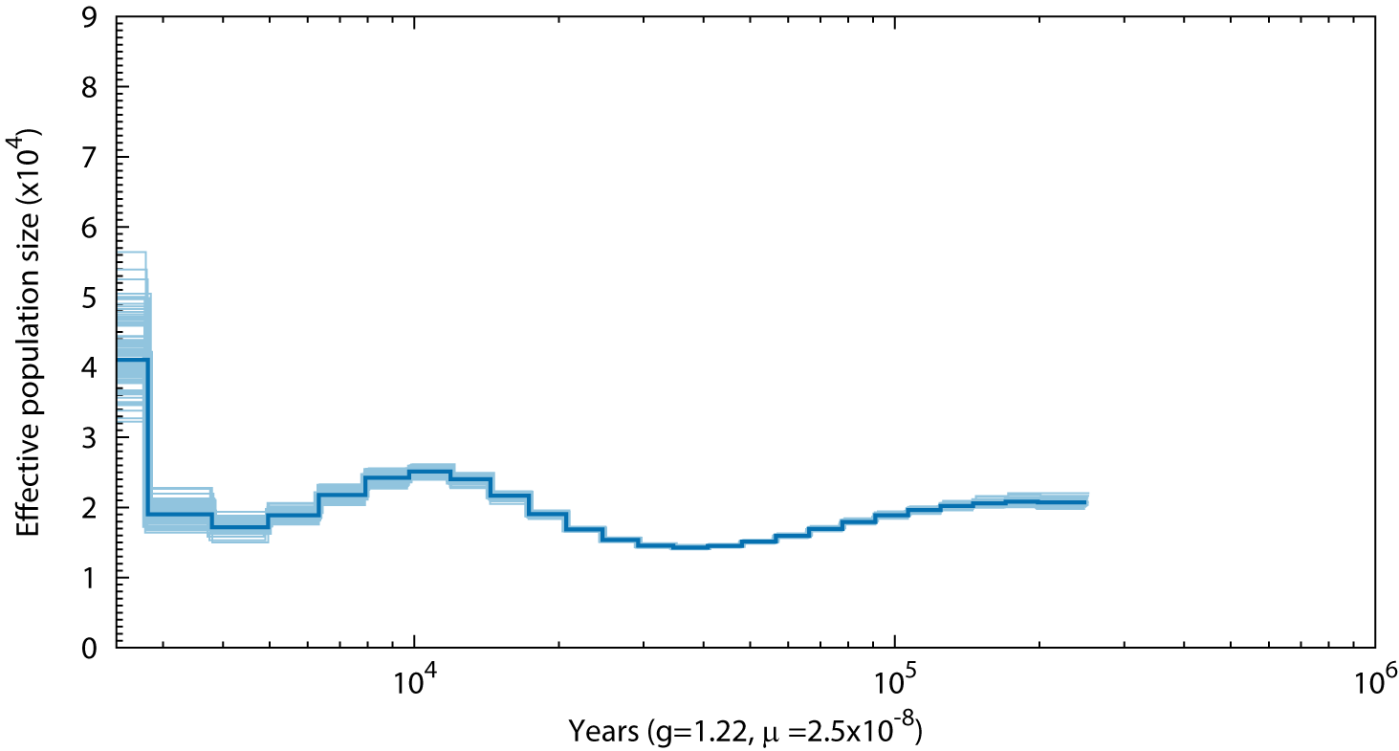


**S5 Demographic History Analysis and PSMC Effective Population Size Estimates for the Scaled Quail (*Callipepla squamata*) v1.0.** Historic estimates of effective population size are presented on the y-axis. The x-axis represents years before present, on a log scale. Generation intervals of 1.22 years were used for the scaled quail (See Methods). Given the absence of any known per-generation *de novo* mutation rates for the scaled quail and the bobwhite, we used the two human mutation rates (μ) of 1.1×10−8 and 2.5×10−8 per generation (See Methods). Darker lines represent population size inferences, while lighter, thinner lines represent 100 bootstraps to quantify the uncertainty of the inferences.
